# Management of a Sixth-Time Reoperation for Aortic Valve Disease With Aortic Pseudoaneurysm Invading the Sternum

**DOI:** 10.1016/j.jaccas.2025.103517

**Published:** 2025-06-04

**Authors:** Ryaan El-Andari, Mansour Alomran, Roderick G.G. MacArthur

**Affiliations:** a Division of Cardiac Surgery, Department of Surgery, University of Alberta, Edmonton, Alberta, Canada; b Division of Cardiac Surgery, King Abdulaziz University for Health Sciences, Riyadh, Saudi Arabia

**Keywords:** aortic valve, circulatory arrest, reoperation

## Abstract

**Objectives:**

Reoperative cardiac surgery is technically demanding. We describe the case of a 57-year-old woman found to have an ascending aortic pseudoaneurysm invading the sternum at the time of her sixth cardiac reoperation.

**Key Steps:**

In cases of considerably challenging sternal re-entry, the following steps are imperative: 1) peripheral establishment of cardiopulmonary bypass; 2) systemic cooling; 3) circulatory arrest followed by sternotomy; and 4) rapid establishment of cerebral perfusion.

**Potential Pitfalls:**

The proximity and adherence of mediastinal structures to the posterior table of the sternum is of paramount significance when considering sternal re-entry. Completion of the sternotomy prior to systemic cooling and circulatory arrest carries an extremely high risk for exsanguination and mortality.

**Take-Home Messages:**

This case describes the operative management of a patient undergoing a sixth sternotomy with an aortic pseudoaneurysm invading the sternum. Although challenging, this case could be performed successfully with careful attention to preoperative imaging, planning, and team communication.

## Visual Summary

**Figure:**
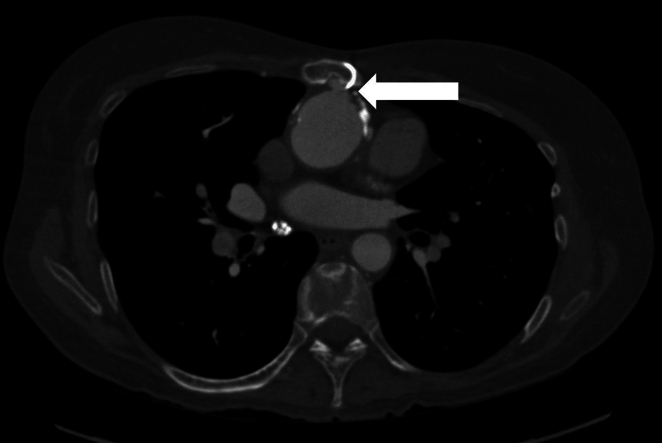

Reoperative cardiac surgery is technically demanding and a significant challenge in cardiac surgery.^1^ Although adherence of structures to the sternum is common, erosion of an aortic pseudoaneurysm into the sternum is a rare occurrence and has been described only in patients with 1 previous cardiac operation.^2^, ^3^, ^4^ Herein, we describe the case of a 57-year-old woman found to have an ascending aortic pseudoaneurysm invading the sternum at the time of a sixth cardiac reoperation, which has not been reported in the literature.

**Take-Home Messages**

• Multiple previous operations result in significant adhesions and more challenging re-entry, which may manifest as a pseudoaneurysm invading the sternum, making sternal re-entry treacherous.
• Imaging, preoperative planning, and team communication are key for safe sternal re-entry and complex reoperation.

## Case Summary

A 57-year-old female patient with 5 previous cardiac surgical procedures presented for a sixth sternotomy for moderate prosthetic aortic valve (AV) stenosis and regurgitation with declining left ventricular(LV) ejection fraction. The patient provided written informed consent to publish this case.

## Past Medical History

The patient’s medical history included coarctation repair at 6 years of age, AV repair at 10 years of age, AV repair and LV outflow tract (LVOT) patch enlargement at 26 years of age, AV replacement (AVR) with a 21-mm bioprosthesis at 29 years of age, and AVR with a 19-mm bioprosthesis at 42 years of age. Since her last surgery, she had been asymptomatic, with prosthetic valve degeneration identified on follow-up imaging.

## Investigations

**Figure 1:**
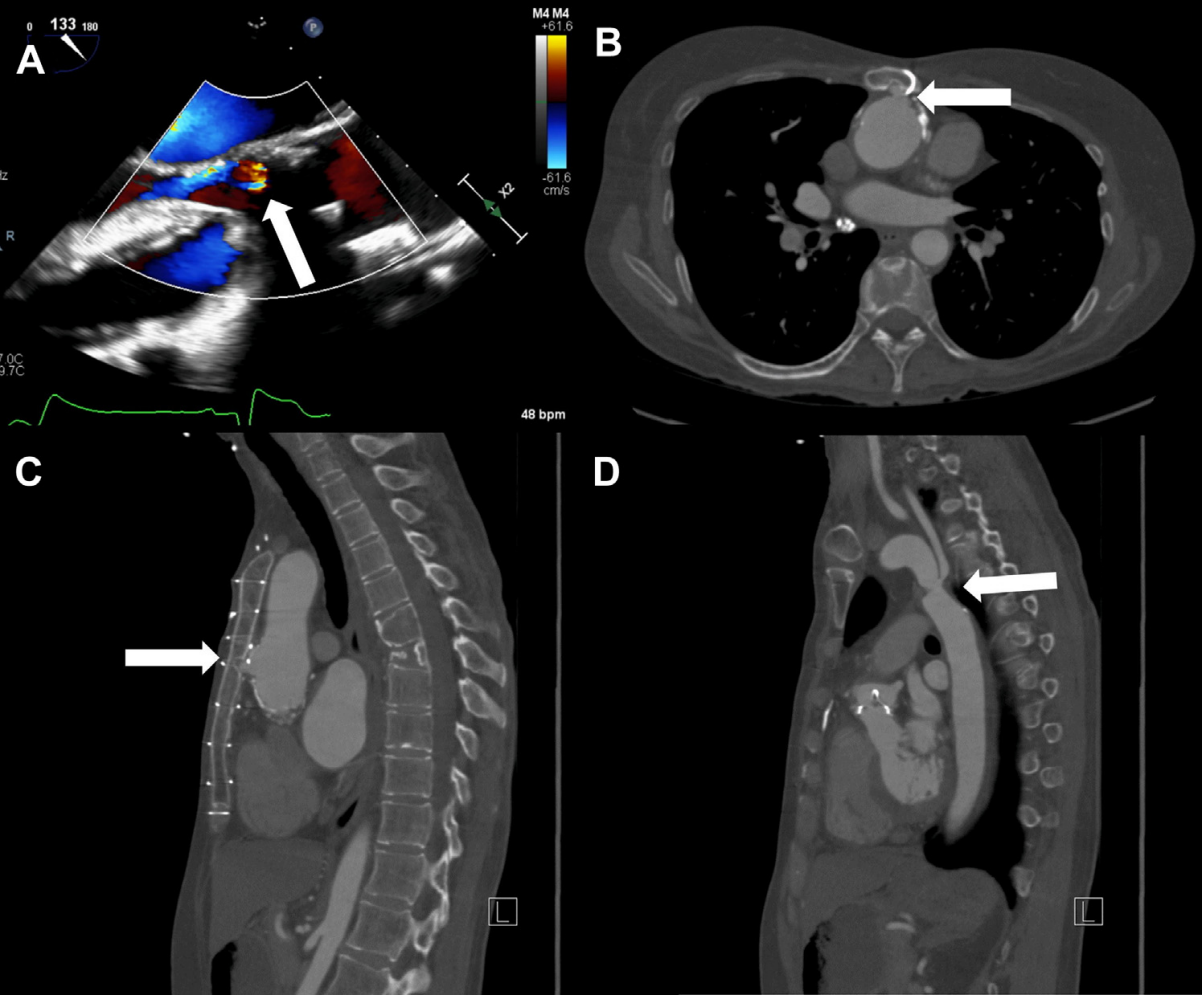
Preoperative Echocardiogram and Computed Tomography Scans Preoperative imaging with echocardiography demonstrating moderate aortic insufficiency (A). Computed tomography demonstrating an aortic pseudoaneurysm invading the sternum (B, C) and a stenotic segment of the descending thoracic aorta (D).

Transthoracic echocardiography demonstrated a prosthetic AV mean gradient of 25 mm Hg, peak velocity of 3.1 m/s, AV area of 1.1 cm^2^, moderate AV insufficiency, and a declining LV ejection fraction of 45% to 50% from 55% to 60% 1 year prior (Figure 1A).

Preoperative computed tomography revealed an aortic pseudoaneurysm invading the posterior table of the sternum (Figures 1B and 1C) and a stenotic descending thoracic aortic segment with a vertebral artery takeoff just proximal to the stenosis (Figure 1D).

## Procedural Steps

### Case setup and cardiopulmonary bypass

The patient was brought to the operating room for AVR and ascending aortic replacement. A cutdown was performed on the left femoral vein and right axillary artery to expose the vessels.

**Figure 2:**
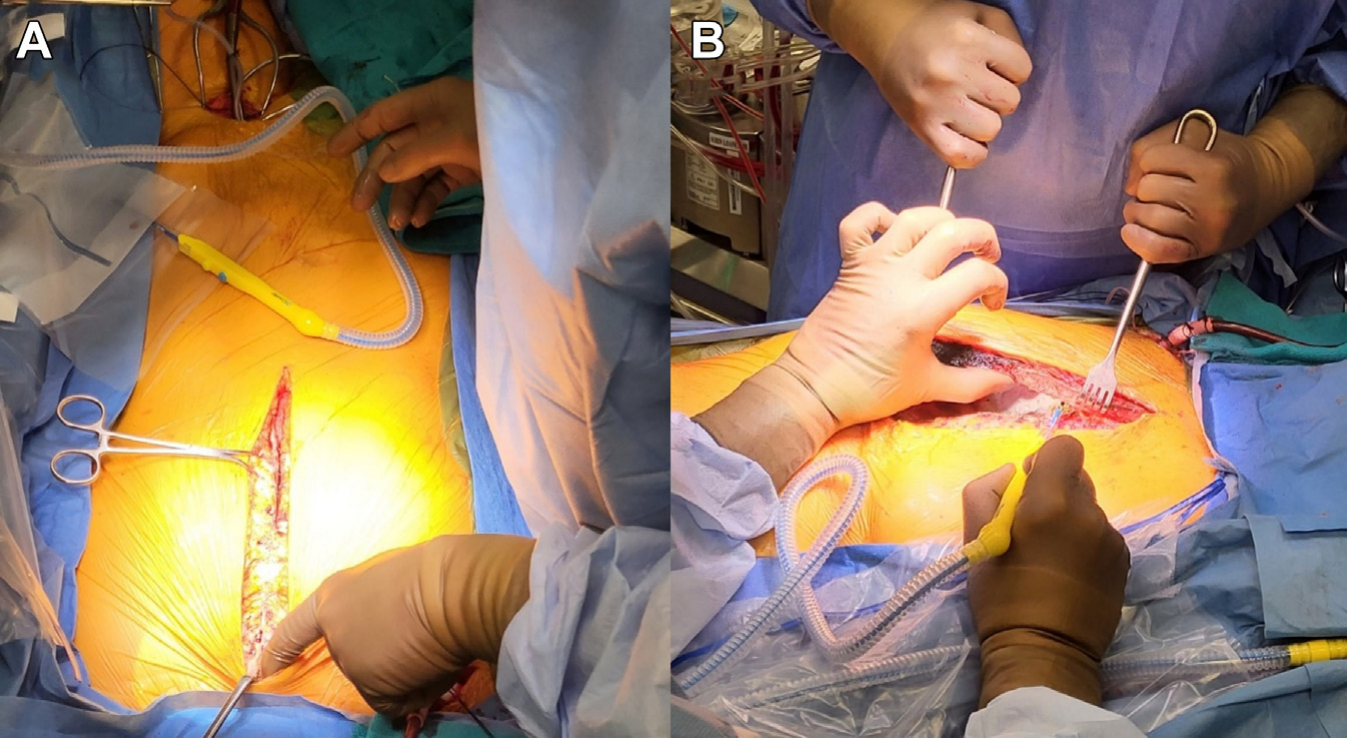
Intraoperative Images of the Sternotomy Intraoperative dissection of the inferior aspect of the sternum up to the level of the pseudoaneurysm (A) and dissection of the posterior table of the sternum following completion of the sternotomy (B).

A median sternotomy skin incision was made. All sternal wires were removed, with the exception of the figure-of-8 wire next to the pseudoaneurysm eroding into the posterior cortex of the sternum (Figure 2A). Dissection of the mediastinal structures away from the posterior table of the sternum proximal and distal to the pseudoaneurysm was performed using electrocautery. Heparin was administered, and a side-biting clamp was placed on the right axillary artery. An arteriotomy was performed, and an 8-mm Gelweave graft (Terumo) was attached via end-to-side anastomosis and cannulated with a 20-F aortic cannula. The left femoral vein was cannulated with a multiport femoral venous cannula up to the superior vena cava. Cardiopulmonary bypass was established, and cooling commenced. We planned a target cooling time of 45 minutes and a target blood temperature <20 °C. The heart fibrillated at low temperature with LV distention, as expected. LV venting was not pursued in this case, as cooling was initiated prior to sternotomy, and the dense adhesions from her previous surgical procedures precluded a safe thoracotomy approach to place an LV vent. The combination of cooling and fibrillation was believed to provide sufficient myocardial protection for the time the heart would be distended prior to circulatory arrest.

### Sternotomy

Once cooled, the patient was placed in the Trendelenburg position, cardiopulmonary bypass was discontinued, and the patient was drained of blood to allow a safer sternal re-entry. The sternotomy was completed. Mediastinal structures were carefully separated from the posterior table of the sternum to allow the placement of a sternal retractor (Figure 2B). A defect in the ascending aorta was identified, and the pseudoaneurysm was found to involve the suture line between the previous graft and aorta.

### Cerebral perfusion

The innominate artery was isolated and clamped to allow antegrade cerebral perfusion. The absolute circulatory arrest time was 19 minutes, with additional arch reconstruction time of 34 minutes, with antegrade cerebral perfusion provided.

### AVR and ascending aortic replacement

The aorta was excised from the sinotubular junction level to the base of the innominate artery. A 26-mm Gelweave graft was anastomosed with running 4-0 Prolene (Johnson & Johnson). The aortic arch was deaired. The ascending graft was clamped and cardiopulmonary bypass resumed. Cardioplegia was administered directly down both coronary ostia.

Dissection to the right superior pulmonary vein was extremely difficult because of this patient’s pericardial window, placed in 1975. However, we were able to isolate the right superior pulmonary vein and placed a left atrial vent. A retrograde cardioplegia catheter was placed into the coronary sinus, with retrograde cardioplegia administered every 30 to 40 minutes during the cross-clamp. Attention was turned to the AV. The valve was inspected, and the leaflets were found to be restricted in motion with poor coaptation centrally. The previous AV prosthesis was excised. A 19-mm Perimount Magna Ease tissue AV (Edwards Lifesciences) was selected for the replacement. The patient had managed well with this valve size for the past 15 years, and her current LVOT was restricted secondary to multiple prior LVOT debridements and surgical procedures, preventing further enlargement.

The proximal aortic anastomosis was extremely difficult because of calcification of aorta anteriorly and calcification of the prior aortic Dacron patch. Following reperfusion, protamine was administered and all cannulas were removed.

The patient was extremely coagulopathic. Three hours of packing were required to achieve mediastinal hemostasis. A Gore-Tex patch (W.L Gore & Associates) was placed over the ascending aortic graft and secured in 4 quadrants. The chest was closed in standard fashion. Postoperative transesophageal echocardiography revealed unchanged biventricular performance and normal prosthetic AV function. The patient was then transferred to the cardiovascular intensive care unit.

### Follow-up

Once in the cardiovascular intensive care unit, the patient experienced a seizure that resolved with levetiracetam. Head computed tomography did not reveal acute changes, and the patient was treated with a 7-day course of levetiracetam. The postoperative course was otherwise unremarkable, and the patient was discharged home on postoperative day 9 in stable condition. Seventeen months later, the patient is alive and well.

## Potential Pitfalls

Reoperation carries increased risk secondary to adhesions, degenerated prosthetic material, and the potential for the development of abnormal anatomy.^1^ Furthermore, each subsequent reoperation adds additional challenges with loss of access to cannulation sites and more difficult sternal re-entry. In this case, the patient was undergoing her sixth cardiac surgery, and the procedure was complicated by difficult adhesions, a small LVOT, and an aortic pseudoaneurysm invading the sternum. Without the establishment of cardiopulmonary bypass, systemic cooling, and circulatory arrest prior to sternotomy, the risk for life-threatening exsanguination was extremely high because of the likely injury to the aorta. A consequence of cooling prior to the sternotomy was the risk for fibrillation prior to placing the LV vent. Although several options are available for LV venting, such as the placement of vents in the right superior pulmonary vein or pulmonary artery following sternotomy, or the placement of an LV apical vent via thoracotomy prior to completion of the sternotomy (Table 1), these approaches were not safe or feasible in this case. Accepting distension of the LV for a limited time while the heart was cool and fibrillating, both of which reduce myocardial oxygen demand and reduce the risk for myocardial injury, was necessary in this case. Although low in this scenario, there is a risk for LV dysfunction with prolonged distension of the ventricle that must be considered. In cases such as this, in which LV venting may be necessary prior to sternotomy, a left apical vent via thoracotomy would be required. Cooling allowed complete circulatory arrest, although cerebral protection is a significant concern, as there is limited time until significant cerebral injury occurs. The challenging sternal re-entry and considerable adhesions increased the time taken to access the innominate artery following sternotomy and consequently time taken to establish cerebral perfusion. All efforts must be made to reduce the time between circulatory arrest and the establishment of cerebral perfusion to reduce the risk for brain injury in this setting.

**Visual Summary:**
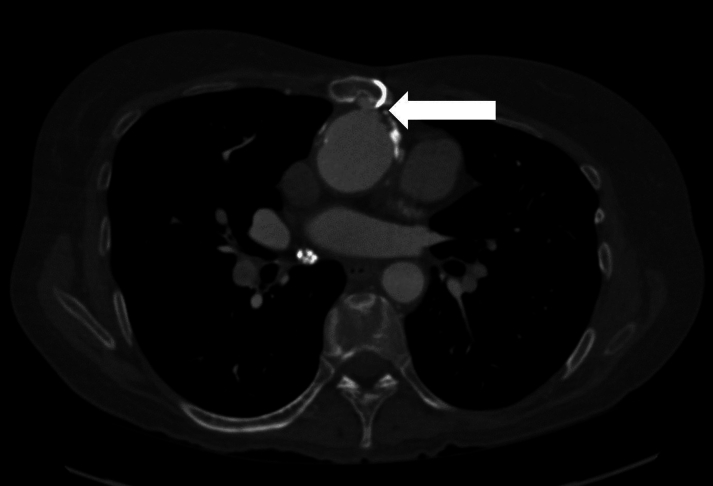
Computed Tomography Scan Demonstrating an Aortic Pseudoaneurysm Invading the Sternum

**Table 1:** Summary of Left Ventricular Venting Strategies During Cardiopulmonary Bypass

Venting Strategy	Pros	Cons
Right superior pulmonary vein	• Provides direct drainage of the LV	• May be difficult to access in some patients • Requires access following sternotomy
Pulmonary artery	• Easily accessible following sternotomy	• Incomplete drainage of the LV • Requires access following sternotomy
Apical vent	• Can be placed prior to completion of sternotomy • Directly drains the LV	• More technically challenging • Increased risk of injury to the LV in reoperative cases

LV = left ventricle.

## Conclusions

Several factors contributed to the success of this case and are translatable to other difficult reoperations. First, detailed preoperative imaging is imperative to understanding the patient’s anatomy.^5^ Second, the development of a detailed plan with clear communication with the various team members is essential for smooth progression of the case.^5^ In this case, the initiation of cardiopulmonary bypass and circulatory arrest before completing the sternotomy prevented potentially life-threatening exsanguination. Planning for future reoperation is crucial, especially for younger patients. The placement of a Gore-Tex patch over the ascending aorta reduces the risk for adherence to the sternum and may help mitigate future risk if reoperation is required. In cases such as this, there is consideration of both cerebral and myocardial protection. Total circulatory arrest allowed safe re-entry and antegrade cerebral perfusion for cerebral protection. Cooling provided myocardial protection until antegrade and retrograde cardioplegia could be administered.

This case describes the operative management of a patient undergoing a sixth sternotomy for AVR and ascending hemiarch replacement with an aortic pseudoaneurysm invading the posterior sternum. Although challenging, this case could be performed successfully with careful attention to preoperative imaging, planning, and team communication.

## Funding Support and Author Disclosures

The authors have reported that they have no relationships relevant to the contents of this paper to disclose.

## References

[1] Bianco V., Kilic A., Gleason T.G. (2020). Reoperative cardiac surgery is a risk factor for long-term mortality. Ann Thorac Surg.

[2] Hasan S.B., Khan F.W., Hashmi S. (2019). Repair of ascending aortic pseudoaneurysm eroding through the sternum. Asian Cardiovasc Thorac Ann.

[3] Akgun T., Kahveci G., Biteker M. (2009). Retrosternal pseudoaneurysm eroding the sternum in a patient six years after aortic valve replacement. J Card Surg.

[4] Caceres M., Halpern D.E., Hooker R.L. (2017). Ascending aortic graft pseudoaneurysm with sternal erosion: a presentation of candidal infection. Ann Thorac Surg.

[5] Hage A., Hage F., Guo L. (2023). Reoperative cardiac surgery in adults: how i teach it. Ann Thorac Surg.

